# Use of Eye Tracking for Assessment of Electronic Navigation Competency in Maritime Training

**DOI:** 10.16910/jemr.12.3.2

**Published:** 2019-07-30

**Authors:** Oguz Atik, Omer Arslan

**Affiliations:** Dokuz Eylul University Maritime Faculty, İzmir, Turkey; Dokuz Eylul University Maritime Faculty, İzmir, Turkey

**Keywords:** Eye movement, eye tracking, region of interest, individual differences, electronic navigation, maritime education, simulation and training, competency assessment

## Abstract

The purpose of this study is to experiment an assessment method using eye tracking technology in simulator based electronic navigation training of ship officers who play a critical role in maritime accidents. The maritime industry focuses on human factor developing and improving regulations, training requirements and technology to prevent marine casualties. The mandatory use of simulations in maritime training as per international regulations includes competency assessment as a vital process. The study involves capturing and analyzing eye movement data from ship officers with sea experience in simulation exercises for assessing competency. A system including an eye tracking analysis software and eye tracking glasses is used for the study. Inferential and descriptive analysis were both used to validate the results. Significant differences were found between electronic navigation competencies of expert and novice ship officers. The results show that the eye tracking technology is a valuable tool for assessment of electronic navigation competency. Comparing novice and expert ship officers’ data proves that eye tracking provides in-depth data which is not obtainable by the available observation methods used in simulation training. The findings show that eye tracking provides the assessor novel data, such as focus of attention, which enables evaluation of the cognitive process and competency. The study, therefore, contributes to maritime education aiming to improve the effectiveness of simulator based maritime training which is vital for maritime safety. It also contributes to scientific research on eye movement in maritime field by proposing the integration of eye tracking in competency assessment in electronic navigation training as a part of simulation based maritime education.

## Introduction

The purpose of the study is to develop an innovative approach to human performance and error research, which play a critical role in maritime accidents and potentially contribute to maritime industry and safety. The research proves that the main contributing factor to marine casualties is human error, which accounts for 75 to 96% of various types of accidents: 84 to 88% of tanker accidents, 79% of towing vessels groundings, 89 to 96% of collisions, and 75% of fires and explosions [1]. Variations in situation awareness levels of the seafarers during critical tasks is frequently linked to human error [2]. The maritime authorities and the industry rely on improving regulations and developing electronic navigation technologies and automation for preventing marine casualties [3]. Automation technology in ship management and operations, such as navigation, engine control, and cargo handling, is constantly increasing. Integrated bridge system (IBS) with minimum manning on the bridge, and unmanned engine control rooms dominate the modern ship operations [4]. Research shows that the approved standard number of bridge equipment increased from 22 to 40 between 1990 and 2006. Integrated bridge equipment provides the navigator with much more information that is much easier to obtain than scattered data from individual navigation aids [5]. Automation is constantly increasing to support seafarers and to overcome the fluctuations in situational awareness caused by factors such as workload, fatigue, and lack of technical and non-technical skills [6]. Besides many advantages of the modern automated systems, overreliance on them creates disadvantages, such as a decrease in situation awareness [7]. Training and competency assessment of maritime officers in realistic simulators for improving automation familiarization and situation awareness is critical for maritime safety.

International Convention on Standards of Training, Certification and Watchkeeping (STCW) for Seafarers includes regulation guidelines on simulator based maritime training such as Bridge Resource Management, Engine Resource Management, and Electronic Navigation. The convention clearly and strictly requires specific training methods, such as competence-based training, as well as competency assessment of seafarers [8]. The 2010 Manila Amendments to STCW Convention and Code requires that competencies in both technical and non-technical skills be demonstrated by ship officers. Approved training ship experience, approved in-service experience, and approved simulator training are the suggested methods by STCW Code for demonstrating competency [8].

The focus of this study is the assessment of electronic navigation skills of ship officers in competency-based simulator training. The study proposes the use of eye tacking technology as an assessment tool to enhance effectiveness of simulation training. The conventional assessment methods used in simulation training are limited to monitoring of the participants from inside an instructor station intentionally separated from the simulation room where the participants complete given tasks. The rooms are separated to create a realistic environment. At most, cameras and microphones are used to improve monitoring capabilities and live observation of mouse tracking provides additional data. However, the process is very limited in comparison to what data eye tracking provides. The conventional methods of observing and monitoring do not provide data to the assessor on focus of attention, which allows evaluation of the cognitive process. Integrating the method proposed in this study in maritime training using the measurements collected from a novice maritime cadet or a course trainee’s performance using eye tracker after completion of a training would help determine the level of competency by comparing eye tracking measurements to the expert benchmark that the instructor establishes. This study aims to prove the importance and novelty of eye tracking by testing the system on two common simulator based electronic navigation training exercises.

The acronyms used are listed in Table 1.

**Table 1:** List of Acronyms

**Acronym**	**Explanation**
AOI	Area of Interest
AIS	Automatic Identification System
BRM	Bridge Resource Management
EBL	Electronic Bearing Line
ECDIS	Electronic Chart Display and Information System
EOG	Electro-OculoGraphy
ERM	Engine Resource Management
IBS	Integrated Bridge System
IMO	International Maritime Organization
POG	Photo-OculoGraphy
RADAR	Radio Detection and Ranging
STCW	International Convention on Standards of Training, Certification and Watchkeeping
VOG	Video-OculoGraphy
VRM	Variable Range Marker

### Competency Assessment in Maritime Training

The effectiveness of education can be measured by learning outcomes [9]. According to Shepard [10], assessments contribute to learning and understanding. Assessment provides valuable feedback and enables measurement of the student’s learning [11]. There is a wide range of literature on assessment techniques. According to Cross [12] [13], every training requires a specific assessment method. Since maritime education is strictly regulated by the STCW Code, competency assessment in simulator based maritime education is well described in the IMO Model Course guidelines. In IMO Model Course 6.10 “Train the Simulator Trainer and Assessor” guidelines, assessment is described as a necessity to enhance the learning process and is critical for certifying the competency of the learner. Assessment is defined as “verification of competency of learners”. The purpose of assessment in a competency based assessment system is to collect sufficient evidence that trainees can perform or behave according to a specified standard in a defined role [14, p.97]. According to Anderson and Krathwohl [15], in their revision of the Bloom’s Taxonomy, there are three domains effecting the assessment method, which are cognitive, psychomotor, and affective. In IMO Model Course 6.10, cognitive is described as things that the learner should know, psychomotor as the skills the learner should be able to do, and affective as the way the learner feels or modifies his/her attitudes. The assessment of cognitive, psychomotor, and affective domains is an essential part in a competence-based system, and evidence of performance needs to be monitored and measured using structured criteria which have to be “relevant, valid, reliable, consistent and realistic” [14, p.97].

At this point, it is important that the simulator can reflect real events. Ability to control, record and play the scene for evaluation and debriefing are important features of the simulators. Observing, monitoring, and recording the activities of the trainee are essential steps in simulator training highlighted in the STCW Code section A-I / 12 [8]. Eye tracking, as a valuable research tool for observing interaction of people with visual information [16], is used in this study in observing, monitoring, and recording activities in simulator training as an assessment tool. Eye tracking provides the assessor the ability of recording the trainee’s gaze, fixation, and attention as well as live observation of the trainee’s activities during a simulation scenario. The traditional observation methods, which are currently used in simulator based maritime training, are limited in collecting objective behavioral data that, according to Hasan et al. (2008), as quoted by [17], can be obtained by capturing the eye movement patterns of a person using the eye tracking technique.

### Eye Tracking Technology

Eye tracking is the method used to measure eye movements and point of gaze with special equipment commonly called an “eye tracker”. There are four common methods used to measure eye movements, including the use or measurement of Electro-OculoGraphy (EOG), Scleral contact lens/search coil, Photo-OculoGraphy (POG) or Video-OculoGraphy (VOG), and Video-based combined pupil and corneal reflection [18] [19] [20].

While the Electro-OculoGraphy (EOG) method relies on measurement of the skin’s electric potential differences, Scleral contact lens/search coil relies on an optical or mechanical reference object mounted on a contact lens. Photo-OculoGraphy (POG) or Video-OculoGraphy (VOG) includes many eye movement recording techniques for measurement of distinguishable features of the eyes [20] [21] [22].

Video-based combined pupil and corneal reflection tracker, which is the most basic form of eye tracking methods, determines the focal points in the visual field with fixations and saccades using cameras and other hardware [18] [20]. Some measurements such as pupil diameter, frequency of blinks, duration of blinks, and number of blinks are also used for deeper analysis of cognitive processing and stress in this system [23].

The most commonly used eye tracking measure-ments according to Sharafi et al. [24], Nivvedan [19] and Lupu & Ungureanu [25] are;

- Fixation, which is the time taken for processing image by fovea,
- Saccade, which is the time interval between two fixations,
- Scan path, which is the spatial arrangement of a sequence of fixations,
- Gaze duration, which is the cumulative duration and average spatial location of a series of consecutive fixations within an area of interest.

The usability of the eye tracker is appraised by metrics that are relevant to the duties and their inherent cognitive activities. For example, distribution of fixation was used as a measurement of mental workload by Nocera et al. [26] [27], whereas fixation time was used to determine importance of giving the eye time to actually look for objects in the surroundings by Hareide & Ostnes [28].

Researchers focus on eye tracking technology in variety of disciplines, such as the medical field [29] [30], marketing [31], usability research [32], information technologies [33], agriculture [34], multimedia technology [17] [35], education [36] [37], and aviation [38]. Review of human factor researches in marine transportation field shows that a very limited number of studies have been carried out on eye tracking for ship officers. Kum, Furusho, & Arslan [39] collected fixation data from maritime cadets on bridge simulator using an “eye mark recorder” and obtained findings on relationship between experience and focus of attention. Lutzhoft & Dukie [40] studied focus of attention and fixation of ship officers during watchkeeping, aiming to contribute to safe navigation. In the study conducted by Forsman et al. (2012) [41], the behavior of novice and expert boat drivers have been tested during high speed navigation at sea. Gaze behavior from both novices and experts was investigated with respect to direction, object and distance of fixations. Muczynski & Gucma [42] used eye tracking for their research on the human factor in marine operations. Hareide & Ostnes [43] tested use of eye tracking technology in marine transportation focusing on Integrated Bridge System and human-machine interaction. Di Nocera et al. [44] studied fatigue and attention using eye trackers in simulators. Thus, this study presents a novelty emphasizing the potential contribution of eye tracking technology in simulator based maritime training and competency assessment required by STCW.

## Methods

A total of 33 recordings on radar (radio detection and ranging) and ecdis (electronic chart display and information system) simulation scenarios were captured from 17 oceangoing deck officers consisting of 10 masters, 2 chief officers, and 5 watchkeeping officers. The average recording was 5,34 minutes long for the ecdis experiment and 5,68 minutes long for the radar experiment. Six of the participant officers who previously received a training course on the ecdis and radar used in the experiment were familiar with the system and were recorded as experts. The rest of the participants who were tagged as novices had experience with radar and ecdis since they worked onboard ships. However, they were not familiar with the system and the specific brands used for the experiment. This research complied with the American Psychological Association Code of Ethics and informed consent was obtained from each participant.

Radar and ecdis were selected for the study because they are the two main components of bridge navigation, which require training certification within the scope of STCW to be used by the ships’ deck officers [45] [46]. It is important to emphasize to compare the two that the radar, which has been available on merchant ships since 1944 [47; p.1] is much simpler than ecdis which was mandated only in 2008 considering functions and operation. The radar is a navigational equipment which assists in safe navigation and in avoiding collision by indicating, in relation to own ship, the position of other surface craft, obstructions, and hazards regarding navigation objects and shorelines [47; p. 457]. Ecdis is an electronic chart display and information system, which is an example of a geographical information system (GIS) that has a database of geographical information that can be filtered and arranged in a display for the convenience of the user. International Maritime Organization Ecdis standards came into force in 1996 and it was then possible for a maritime vessel to replace its paper nautical charts with an ecdis system [47; p.328-329].

**Figure 1:**
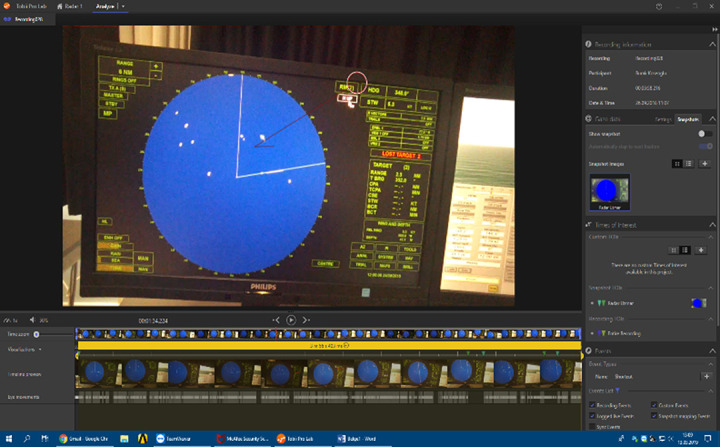
A screenshot of a participant’s recording.

The eye tracking data was collected using Tobii Pro Glasses 2 (gaze sampling frequency 100 Hz), which were calibrated for each participant before recordings, and analyzed using Tobii Pro Lab Analyzer software to obtain metric and visual data. Figure 1 shows a recording of a participant performing on the radar experiment.

**Figure 2:**
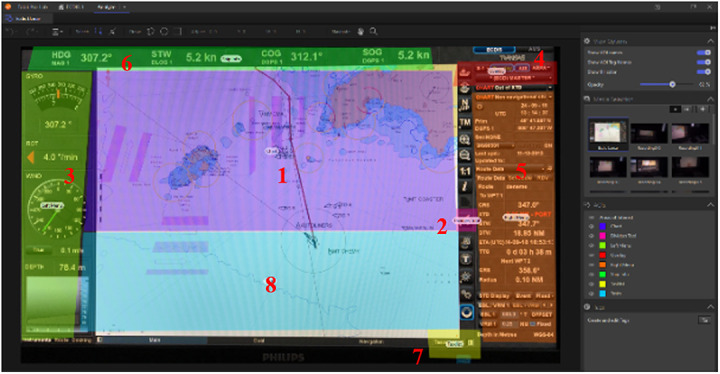
Areas of interest on the ecdis display.

**Figure 3:**
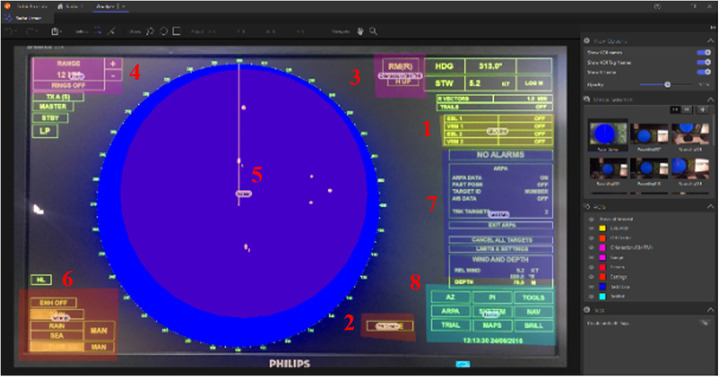
Areas of interest on the radar display.

A radar simulation scenario was created involving completion of different tasks using functions, such as electronic bearing line (EBL), variable range marker (VRM), off center, picture orientation, range, and settings, which were also designated as areas of interest (Figure 3). The ecdis simulation scenario involved using charts menu, dividers, overlay function, ship info function, checking different parts of the menu, and a task list function, which were also assigned as AOI (Figure 2).

Each participant was given a written task list to be completed on the radar and a separate task list for ecdis exercise. The tasks were randomized to avoid any learning effect. Radar and ecdis recordings were captured separately and glasses were recalibrated in between exercises.

During the ecdis exercise the participants were expected to check vessel traffic and course to follow to enter a channel, which involved focusing on the AOI chart and did not require any action, but required an ability to interpret the chart data to get the course information by using the cursor. Another task required obtaining distance information from a specific land mark on the chart, which required the use of dividers, a small tab visible on the main display. Setting the radar and the ais (automatic identification system) overlay on the chart was another task where the participants needed to focus on the overlay button which is at the top right corner of the main display. Checking the display mode, bearing and range information using the variable range marker and electronic bearing line required focusing on the AOI right menu. To obtain course, speed, and waypoint information the participant had to focus on the ship info AOI, which was a horizontal bar at the top of the display. Another task was opening the tasks menu on the display, which is the AOI task list. Task list is a small tab at the bottom right of the display. Checking targets and alarms was another task which required entering the tasks tab and included checking on different submenus. The AOI “left menu” had no use in any of the tasks and the participants did not need to focus on the left menu. The AOI left menu was created to support and validate the study (Table 2).

**Table 2:** List of areas of interest and corresponding tasks for ecdis exercise.

	**AOI**	**Task**
1	Charts	Checking vessel traffic, course to follow
2	Dividers	Obtaining distance information from a specific land mark on chart
3	Left Menu	Had no use in the exercise
4	Overlay	Setting RADAR and AIS overlay on chart
5	Right Menu	Checking display mode, bearing and range information
6	Ship Info	Obtaining course, speed, waypoint information
7	Task List	Opening tasks menu on display
8	Task	Checking targets and alarms menu

The numbers on the AOI figures (Figure 2 and 3) indicate the list of AOI and corresponding tasks (Table 2 and 3).

**Table 3:** List of areas of interest and corresponding tasks for radar exercise.

AOI		Task
1	EBL/VRM	Obtaining distance and bearing information from closest land
2	Off Center	Setting off-center function
3	Orientation	Setting up display orientation north up and relative motion
4	Range	Setting up Radar range
5	Screen	Checking vessel traffic, course to follow
6	Settings	Setting up gain, tune, rain and sea clutter
7	Task Data	Finding the sub-menu information of the selected task
8	Task List	Checking Parallel Index, ARPA, NAV, BRILL functions

During the radar exercise, the participants were required to obtain distance and bearing information from the closest landmark which involved the use of EBL and VRM functions of the radar located at the top right of the display. These are measurement controls used by the cursor. Another task was to use the off-center button which shifts the own ship position to a pre-registered point on the screen. The participants were also asked to switch between north up, course up, head up, true motion, and relative motion, which required focusing on AOI orientation, a tab at the top right of the display. Two buttons on the top left were necessary to change the range of the radar for another task, which was the AOI range. The participants had to focus on AOI to check vessel traffic and the course to follow. Adjusting for gain, tune, rain clutter, and sea clutter required to focus on the settings at the bottom left corner, which was another AOI. The participants were asked to check the target data that is under a submenu and they had to use the push buttons focusing on the AOI task data. AOI task list included the tabs at the bottom right corner of the display and the participants were required to check each tab controlling parallel index, arpa, navigation, and brilliance functions.

Total fixation duration, which is the time the eye remains focusing on an AOI [48], and total fixation count, which is the number of fixations on an AOI [49] were analyzed for the purpose of this study. Fixation duration was used because longer fixation duration can indicate more effortful cognitive processing on an AOI [48] [50]. Fixation count was used because more fixations can be an indication of effort to complete a certain task by a participant [51]. Descriptive and inferential analysis were both used to evaluate the eye tracking data collected. Evaluation of live recordings and visual heat maps were used to support the results. Total fixation duration shows the total amount of time the participant is fixated on a specific AOI while total fixation count shows the total number of fixations on an AOI during the simulation scenario. The non-parametric Mann-Whitney U test was used to test the research hypotheses, which predicted that there would be significant differences between the novice and the expert participants’ fixation duration and fixation count measurements on the ecdis and the radar experiments.

The means for the novice and expert participants’ fixation duration and fixation count data were compared for the purposes of descriptive analysis.

## Results

The Shapiro-Wilk test of normality was conducted for the statistical analysis [52] [53] and the results showed that the conditions for a normally distributed data (p > .05) were not met. A non-parametric two-tailed Mann-Whitney U test on 95% confidence level, which does not require normally distributed data [54] was run to determine the differences between expert and novice participants’ fixation durations and fixation counts on the areas of interest designated for the ecdis and radar exercises.

### Ecdis Experiment

The Mann-Whitney U test run indicated that there are significant differences between novice and expert participants’ fixation duration measurements in AOI chart, left menu, overlay, right menu, and task (Table 4).

**Table 4:** Mann-Whitney U Test Results for total fixation durations of novice and expert participants on ecdis exercise.

AOI	Participant	Mann-Whitney U Test Results
Chart	Novice (Mdn = 57.29) Expert (Mdn = 27.18)	U = 11.00, p = .027, r = 0.54
Dividers	Novice (Mdn = 1.52) Expert (Mdn = 1.76)	U = 31.00, p = .84, r = 0.05
Left Menu	Novice (Mdn = 8.08) Expert (Mdn = 0.41)	U = 6.00, p = .007, r = 0.66
Overlay	Novice (Mdn = 10.45) Expert (Mdn = 2.16)	U = 11.00, p = .027, r = 0.54
Right Menu	Novice (Mdn = 59.21) Expert (Mdn = 17.22)	U = 2.00, p = .002, r = 0.76
Ship Info	Novice (Mdn = 1.44) Expert (Mdn = 0.17)	U = 18.50, p = .14, r = 0.36
Task List	Novice (Mdn = 4.84) Expert (Mdn = 3.84)	U = 29.00, p = .69, r = 0.10
Task	Novice (Mdn = 47.95) Expert (Mdn = 29.84)	U = 12.50, p = .039, r = 0.50

**Figure 4:**
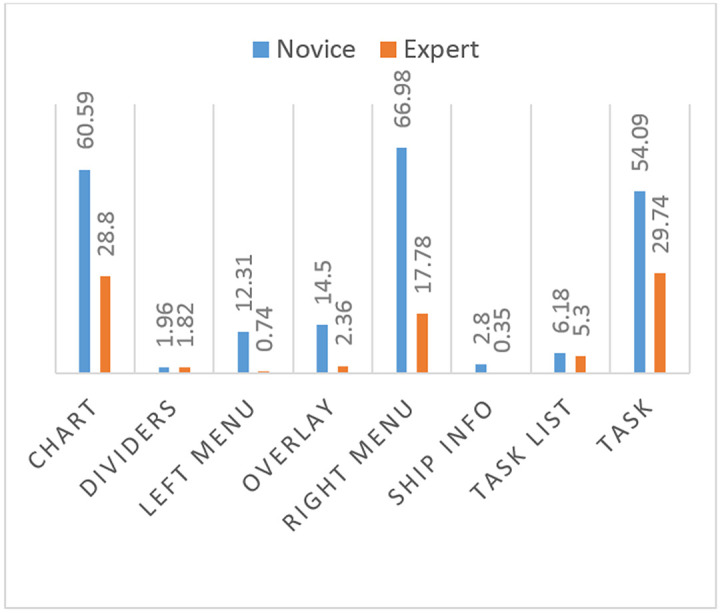
Comparison of means of total fixation duration measurements of novice and expert participants on ecdis.

The means of fixation duration measurements on all areas of interest are larger for the novice participants than for the expert participants (Figure 4).

The Mann-Whitney test showed that there are a significant differences between novice and expert participants’ fixation count measurements in AOI chart, left menu, right menu, and task (Table 5).

**Table 5:** Mann-Whitney U Test Results for total fixation counts of novice and expert participants on ecdis exercise.

AOI	Participant	Mann-Whitney U Test Results
Chart	Novice (Mdn = 135.00) Expert (Mdn = 75.50)	U = 8.00, p = .012, r = 0.61
Dividers	Novice (Mdn = 3.00) Expert (Mdn = 3.00)	U = 31.00, p = .839, r = .049
Left Menu	Novice (Mdn = 30.00) Expert (Mdn = 2.00)	U = 7.00, p = .009, r = 0.64
Overlay	Novice (Mdn = 24.00) Expert (Mdn = 6.50)	U = 14.50, p = .062, r = 0.45
Right Menu	Novice (Mdn = 161.00) Expert (Mdn = 49.50)	U = 2.00, p = .002, r = 0.76
Ship Info	Novice (Mdn = 7.00) Expert (Mdn = 1.00)	U = 21.00, p = .221, r = 0.30
Task List	Novice (Mdn = 11.00) Expert (Mdn = 9.50)	U = 32.50, p = .96, r = 0.01
Task	Novice (Mdn = 159.00) Expert (Mdn = 64.00)	U = 12.50, p = .039, r = 0.50

**Figure 5:**
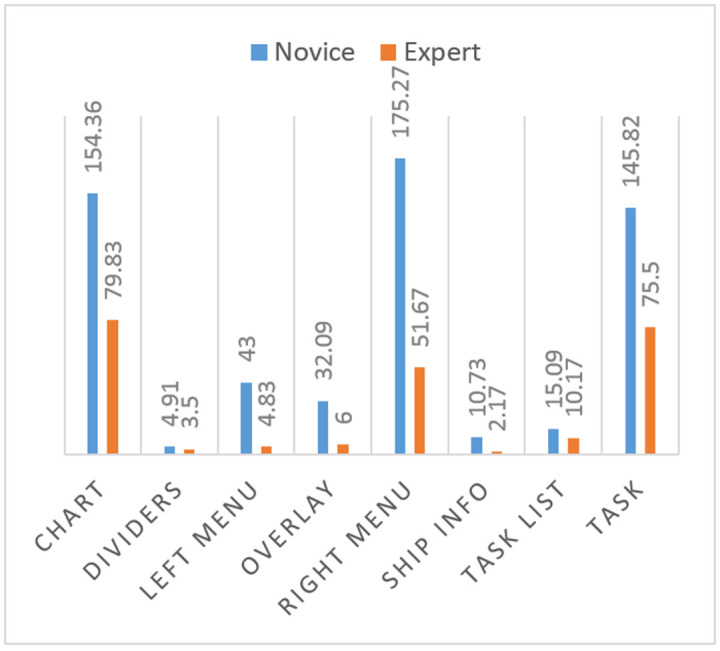
Comparison of means of total fixation counts of novice and expert participants on use of ecdis.

The means of fixation count measurements on all areas of interest are larger for the novice participants than for the expert participants (Figure 5).

### Radar Experiment

The Mann-Whitney U test run indicated that there is a significant difference between novice and expert participants’ fixation duration measurements in AOI “task data” (Table 6).

**Table 6:** Mann-Whitney U Test Results for total fixation durations of novice and expert participants on radar exercise.

AOI	Participant	Mann-Whitney U Test Results
EBL/VRM	Novice (Mdn = 10.42) Expert (Mdn = 3.7)	U = 23.00, p = .448, r = 0.19
Off Center	Novice (Mdn = 0.30) Expert (Mdn = 0.07)	U = 20.00, p = .265, r = 0.28
Orientation	Novice (Mdn = 12.35) Expert (Mdn = 11.10)	U = 29.00, p = .914, r = 0.03
Range	Novice (Mdn = 3.36) Expert (Mdn = 1.90)	U = 16.00, p = .129, r = 0.38
Screen	Novice (Mdn = 82.80) Expert (Mdn = 53.56)	U = 21.00, p = .329, r = 0.24
Settings	Novice (Mdn = 6.08) Expert (Mdn = 6.86)	U = 26.00, p = .664, r = 0.11
Task Data	Novice (Mdn = 59.72) Expert (Mdn = 26.76)	U = 11.00, p = .039, r = 0.52
Task List	Novice (Mdn = 11.36) Expert (Mdn = 6.57)	U = 21.00, p = .329, r = 0.24

**Figure 6:**
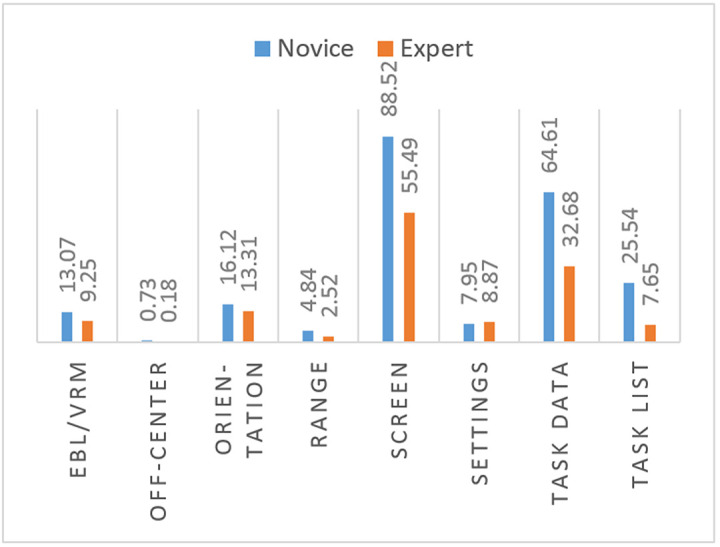
Comparison of means of total fixation duration of novice and expert participants on use of RADAR.

The means of fixation duration measurements on all areas of interest, except setting, are larger for the novice participants than for the expert participants (Figure 6).

The Mann-Whitney test showed that there is a significant difference between novice and expert participants’ fixation count measurements in AOI “task data” (Table 7).

**Table 7:** Mann-Whitney U Test Results for total fixation counts of novice and expert participants on radar exercise.

AOI	Participant	Mann-Whitney U Test Results
EBL/VRM	Novice (Mdn = 26.00)[#break#]Expert (Mdn = 14.00)	U = 19.00,[#break#]p = .232, r = 0.30
Off Center	Novice (Mdn = 1.50)[#break#]Expert (Mdn = 0.50)	U = 20.00,[#break#]p = .263, r = 0.28
Orientation	Novice (Mdn = 16.00)[#break#]Expert (Mdn = 18.00)	U = 27.00,[#break#]p = .744, r = 0.08
Range	Novice (Mdn = 12.00)[#break#]Expert (Mdn = 8.00)	U = 16.50,[#break#]p = .142, r = 0.37
Screen	Novice (Mdn = 185.00)[#break#]Expert (Mdn = 133.50)	U = 22.00,[#break#]p = .386, r = 0.22
Settings	Novice (Mdn = 18.00)[#break#]Expert (Mdn = 15.50)	U = 26.00,[#break#]p = .664, r = 0.11
Task Data	Novice (Mdn = 146.00)[#break#]Expert (Mdn = 70.50)	U = 11.00,[#break#]p = .039, r = 0.52
Task List	Novice (Mdn = 39.50)[#break#]Expert (Mdn = 26.00)	U = 17.50,[#break#]p = .175, r = 0.34

**Figure 7:**
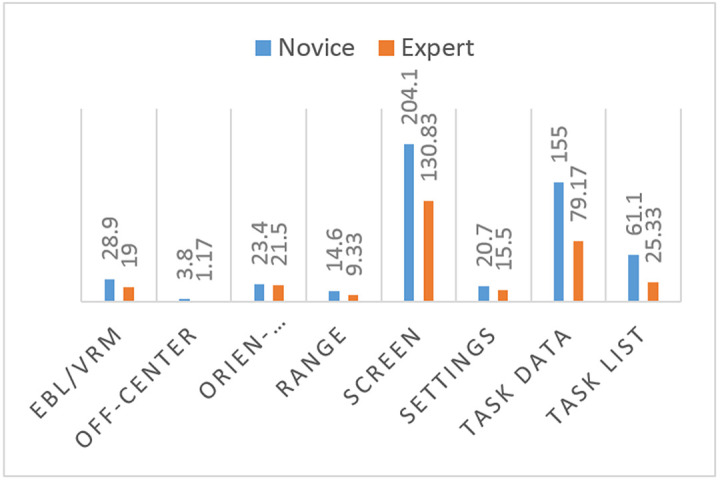
Comparison of means of AOI total fixation count of novice and expert participants on use of radar.

The means of fixation count measurements on all areas of interest are larger for the novice participants than for the expert participants (Figure 7).

## Discussion

This study aims to show the value and usability of eye tracking in electronic navigation competency training as an assessment tool and proposes the integration of the tool in maritime training. The conventional monitoring and observation methods used in simulator based maritime training are limited in assessing the participants’ focus of attention, which is possible to measure with eye tracking. This novice-expert comparison study is designed to prove that the proposed assessment method can be used in competency training where the trainees (novice) are expected to perform at an expert level after completion of training and can be evaluated using eye trackers for competency. In this sense, the novice would be a maritime cadet or, for instance, an ecdis course trainee. The measurements collected from the trainee’s performance using the eye tracker after completion of the training would help determine the level of competency compared to the expert benchmark the instructor establishes.

For the purpose of the study, the statistical results validate the research hypothesis, which predicted that there would be significant differences between the novice and the expert participants’ fixation duration and fixation count measurements on ecdis and the radar experiment.

While there were significant differences in almost all of the AOI measurements in the ecdis experiment and on one of the AOI in the radar experiment, the comparison of the means shows that fixation duration and fixation count measurements on all areas of interest were larger for the novice participants on both ecdis and radar, except the AOI settings on radar on which the experts’ fixation duration measurements were slightly larger.

The Mann-Whitney U test indicated that there are significant differences between novice and expert participants’ fixation duration measurements in AOI chart, left menu, overlay, right menu, and task. The differences in measurements of dividers, ship info, and task list are not significant. As mentioned in the method section, some of the tasks were easier to complete. Dividers AOI was a tab with a dividers picture on it right on the display and the novice had no trouble finding it. Obtaining the course, speed, and waypoint information was also an easier task involving the AOI ship info, a bar on top of the display. Opening the task list, which is a small tab on the bottom right of the display was also an easier task.

There were significant differences between novice and expert participants’ fixation count measurements in AOI chart, left menu, right menu, and task as well. However, measurements on AOI overlay, despite the results of the fixation duration measurements, was not significant. Turning on the radar and ais overlay function involved three buttons at top right of the display, which was a relatively easier task.

Comparing the means of both fixation duration and count measurements in ecdis experiment the novice participants fixated on all areas of interest longer than expert participants, which proves that eye tracking can actually be used as an assessment tool. The novice trainees are expected to reach a level of expert competency at the end of a training and the instructor can test their skills measuring the fixation data. Searching for data unproductively and focusing on redundant information or functions on the display to complete a task on a navigation equipment are indicators of incompetency and can be simply tested by eye tracking. For example, the novice participants fixated on the area of interest left menu significantly, which had no use in the exercise and was set as AOI intentionally by the researchers. None of the tasks involved use of the left menu which included data such as depth of the sea, true wind and relative wind, and the expert participants’ measurement figures on this AOI are very small because they are familiar with the system display. This is valuable information to validate the capabilities of eye tracking as a unique competency assessment tool.

In general, the fixation duration of the novice participants on the areas of interest, which are not under any submenu but rather directly on the main display, are still longer than the expert participants’, but not as much as in the other areas of interest, which are under submenus and more sophisticated. This relates to the complexity and usability of the systems and validates the necessity for training and assessment.

The differences in ecdis performances were much greater than the radar performances, which is expected because the radar became mandatory onboard ships much earlier than the ecdis. The mandatory carriage of ecdis for ships other than High-Speed Craft, which was mandated in 2008, commenced only in 2012, while radar was introduced in 1944. Unstandardized and complicated operation of ecdis with many functions compared to much simpler and standardized radar is another cause of the differences in the results. The operational standardization of ecdis is necessary especially considering many different manufacturers.

The Mann-Whitney U test run indicated that there is a significant difference between novice and expert participants’ fixation duration and fixation count measurements in AOI “task data”. The participants were asked to check the target data which are under a submenu and they had to use the buttons and tabs focusing on the AOI task data, which made the task more difficult reflecting on the results.

While the results on most of the areas of interest are not significantly different on radar, comparing the means of both fixation duration and count measurements, the novice participants fixated on all areas of interest longer than expert participants, except AOI settings, which is the adjustments at bottom left that are standard on most radar equipment. Although the radar is one of the oldest and most standard navigational equipments onboard ships, the difference between the novice and the expert participants indicates importance of system familiarity for the purpose of this study.

**Figure 8:**
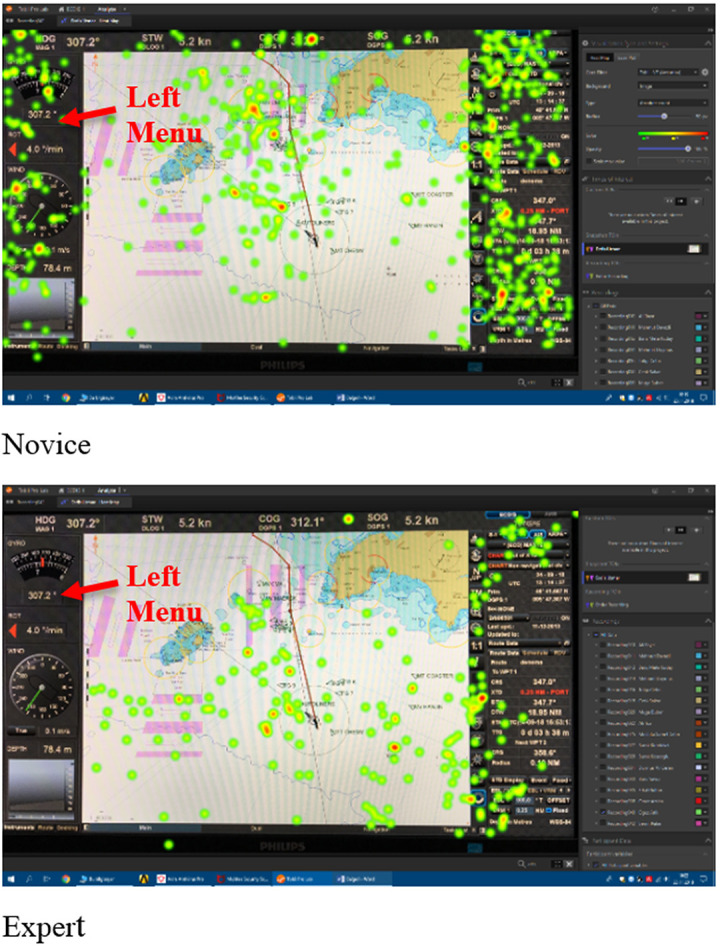
Comparison of sample novice and expert visual heat map data on use of ecdis.

This study focused on metric data. However, a comparison of the heat map visualizations of the novice (top) and expert (bottom) participants’ eye movements (fixations) provided by the Tobii Pro Lab Analyzer software in Figure 8 is given as an example to visualize and support the metric results of the study. The heat maps show how the eye movements are distributed over the image (ecdis display). The visualizations can effectively reveal the focus of visual attention where the color red is the most focused [55].

The visualization data in figure 8 shows that the novice participants’ eye movements are much more scattered on the screen, which indicates that they unconsciously scanned the display seeking for the information and functions to complete the tasks given by the researchers, not knowing where to focus. The expert participants’ eye movements are more grouped which indicates that they focused on the specific parts of the ecdis display knowing exactly where to find necessary information and functions. In the heat maps, it is visible that the novice participants, contrary to expert participants, fixated on all parts of the display some of which were not necessary for the completion of the given tasks such as the AOI left menu.

**Figure 9:**
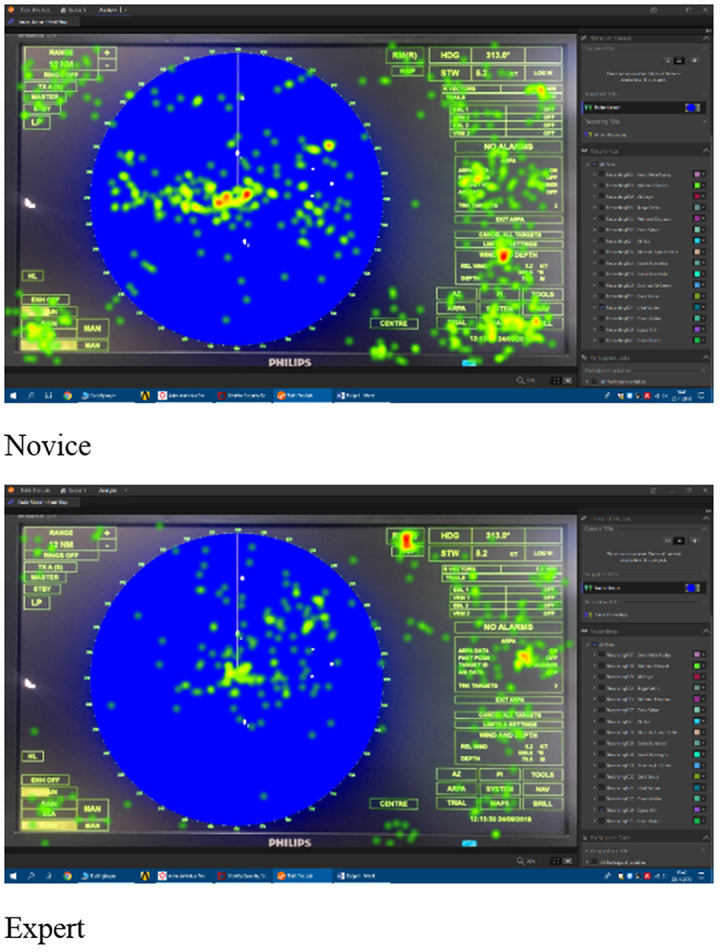
Comparison of sample novice and expert visual heat map data on use of radar.

Similar to the ecdis heat map, on the sample radar heat maps in figure 9, the novice participant’s eye movement pattern is more scattered and the expert is more focused.

## Conclusion

The results show that the eye tracking technology can be a valuable tool for assessment of electronic navigation competency. This study, comparing novice and expert ship officers’ use of electronic navigation aids, proves that at a given task in a simulation scenario eye tracking provides the data of focus and attention of the participants in a way that no other assessment and evaluation method can. Eye tracking, capturing the eye movements of a person, provides the assessor the data of how long, how many times, where, and in what sequence they focused on the display. The conventional “observation” method used by the simulator instructor is very limited in assessment of certain tasks in exercises because of the physical constraints and eye tracking has the potential to fill in that gap.

The major limitation for the eye movement studies and practices in maritime education is the cost of the eye tracking systems. However, considering its potentials it should be integrated into the educational system in maritime institutions. This study proposes the integration of eye tracking method in competency assessment in electronic navigation specifically including ecdis training, which is already mandatory as per STCW.

A key contribution of this study is the introduction of eye movement research in the maritime education field and it shows that its integration in future studies in simulation based maritime training would be truly useful in improving and enhancing effectiveness of maritime education and training. This study also shows that ecdis, which is vital for navigational safety, is a complicated system to operate even for the experienced professionals and usability studies using eye tracking on ecdis would be very helpful in navigation training, considering its critical role in maritime safety. More eye tracking research on situational awareness, stress, and fatigue, which are vital for maritime safety, would be valuable for the industry. This study focused on the use of metric data to prove that eye tracking can be a useful method for assessing electronic navigation competency of an officer, which can very well used for maritime cadets as well. The visual data and the live recording data were also evaluated to confirm the metric data and it is clear that the visualizations are very descriptive and useful assessment tools for the purpose of the study. In future studies, focusing on heat maps and gaze data collected in maritime simulators will also be useful.

## Ethics and Conflict of Interest

The author(s) declare(s) that the contents of the article are in agreement with the ethics described in http://biblio.unibe.ch/portale/elibrary/BOP/jemr/ethics.html and that there is no conflict of interest regarding the publication of this paper.
